# Epigenetic signatures and cellular stress response pathways in metabolic dysfunction-associated steatotic liver disease: a personalized medicine perspective

**DOI:** 10.3389/fphys.2026.1787963

**Published:** 2026-04-30

**Authors:** Fabian M. Cortés-Mancera, Marianne G. Rots, Han Moshage, Johanna C. Arroyave-Ospina

**Affiliations:** 1 Departamento de Ciencias Aplicadas, Grupo de Investigación e Innovación Biomédica, BioMed, Instituto Tecnológico Metropolitano, Medellín, Colombia; 2 Department of Pathology and Medical Biology, University of Groningen, University Medical Center Groningen, Groningen, Netherlands; 3 Department of Gastroenterology and Hepatology, University of Groningen, University Medical Center of Groningen, Groningen, Netherlands; 4 Grupo de Gastrohepatología, Sede de Investigación Universitaria SIU, Universidad de Antioquia, Medellín, Colombia; 5 Departamento de Fisiología y Bioquímica, Facultad de Medicina, Universidad de Antioquia, Medellín, Colombia

**Keywords:** cellular stress response pathways, DNA methylation, epidrugs, epigenetic editing, histone post-translational modifications, metabolic dysfunction-associated steatotic liver disease (MASLD), metabolic stress, mitochondrial dysfunction

## Abstract

Metabolic dysfunction-associated steatotic liver disease (MASLD) is the most common chronic liver disease around the world, with at least 40% global prevalence. Although genetic susceptibility contributes to disease risk, it does not fully explain the marked interindividual variability in disease onset, severity, and progression. Increasing evidence indicates that epigenetic mechanisms act as critical mediators between genetic predisposition and environmental exposures, shaping hepatic stress responses and metabolic dysfunction in MASLD. Epigenetic regulation, including DNA methylation and histone modifications, plays a fundamental role in maintaining metabolic homeostasis and coordinating cellular responses to metabolic, oxidative, and organellar stress, e.g., endoplasmic reticulum stress. Dysregulation of these processes has been consistently associated with hepatic steatosis, inflammation, fibrosis, and disease progression. Moreover, epigenetic control of circadian rhythms and molecular clock pathways further link metabolic imbalance to liver dysfunction and biological aging. Importantly, epigenetic signatures are stable, positioning them as attractive biomarkers for disease diagnosis, prognosis, and patient stratification, but also potentially reversible, making them promising targets for therapeutic intervention. Advances in epigenomic profiling and translational research are increasingly supporting the integration of epigenetic information into personalized medicine approaches for MASLD. In this review, we synthesize current experimental and translational evidence on epigenetic alterations involved in MASLD pathophysiology, with a particular focus on their role in cellular stress response pathways. We discuss the potential of epigenetic signatures as biomarkers and therapeutic targets, highlighting their relevance for the development of precision-based strategies in the management of MASLD.

## 1 Introduction

Metabolic dysfunction–associated steatotic liver disease (MASLD) is currently the most prevalent chronic liver disease worldwide. Recent estimates indicate a global prevalence of approximately 38%, with projections suggesting an increase to over 50% by 2040 (Younossi et al., 2025). MASLD encompasses a broad spectrum of liver disorders characterized by hepatic steatosis in the presence of at least one cardiometabolic risk factor (Rinella et al., 2023). Given its close association with systemic metabolic dysfunction, understanding the underlying molecular mechanisms driving disease initiation, progression, and heterogeneity remains a critical challenge.

The pathophysiology of MASLD is currently explained by a “multiple-hit” model, in which genetic susceptibility and environmental factors interact to promote metabolic dysfunction and liver injury (Arroyave-Ospina et al., 2021; Steinberg et al., 2025). Several genetic variants, including those in PNPLA3, TM6SF2, and MBOAT7, have been consistently associated with increased susceptibility to MASLD and a higher risk of disease progression (Moretti et al., 2024). However, genetic predisposition alone does not fully account for the variability in disease onset, severity, or clinical outcomes.

Beyond genetic factors, multiple metabolic and environmental factors such as insulin resistance, adipose-derived hormones, alterations in the gut microbiota, and lifestyle-related exposure have been implicated in MASLD development (Targher et al., 2025; Huang et al., 2025). These factors exert their effects, at least in part, through epigenetic mechanisms that reprogram gene expression without altering the underlying DNA sequence (Chakraborty et al., 2025). Epigenetic dysregulation, including aberrant DNA methylation patterns and imbalances in histone modifications (such as methylation and acetylation), has been increasingly linked to MASLD pathophysiology (Rodríguez-Sanabria et al., 2022).

Epigenetic regulation plays a fundamental role in maintaining metabolic homeostasis and coordinating cellular responses to metabolic stress (Wu et al., 2024). In addition, circadian rhythms and molecular clock machinery, both critical regulators of hepatic metabolism, are tightly controlled by epigenetic mechanisms (Sato and Sassone‐Corsi, 2022). Disruptions in these processes have been linked to metabolic dysfunction, accelerated epigenetic aging, and an increased susceptibility to liver disease (Sato and Sassone‐Corsi, 2022).

Accumulating evidence indicates that epigenetic marks are not only central drivers of metabolic dysfunction but also hold significant promise as biomarkers for disease diagnosis, prognosis, and patient stratification. Importantly, epigenetic modifications are potentially reversible, making them attractive targets for therapeutic intervention. As such, epigenetic profiling may provide a critical framework for advancing personalized medicine approaches in MASLD and other metabolic disorders.

In this review, we summarize experimental and translational evidence supporting the role of epigenetic signatures in MASLD pathophysiology. We further discuss their potential utility as biomarkers for personalized diagnosis and prognosis, as well as their relevance as therapeutic targets for the development of advanced, precision-based treatment strategies.

## 2 MASLD pathophysiology: general mechanisms and epigenetics

Hepatic steatosis is the hallmark of MASLD development and is initiated by an imbalance in hepatic lipid metabolism, resulting in the accumulation of triglycerides as lipid droplets within hepatocytes. Hepatic lipid droplet accumulation is primarily driven by alterations in three key regulatory mechanisms of lipid metabolism: (1) increased delivery of fatty acids to the liver from dietary sources and adipose tissue lipolysis; (2) enhanced *de novo* lipogenesis; and (3) reduced very-low-density lipoprotein (VLDL) export. Consequently, excess free fatty acids (FFAs), particularly saturated fatty acids (SFAs), trigger lipotoxicity in hepatocytes, leading to mitochondrial dysfunction and endoplasmic reticulum (ER) stress (Arroyave-Ospina et al., 2021). These processes are largely mediated by oxidative stress, which plays a central role in the activation of proinflammatory pathways and ultimately contributes to fibrosis and progression toward end-stage liver disease.

Epigenetic modifications have emerged as central drivers in the establishment of metabolic dysfunction by regulating gene expression across pathways involved in lipid metabolism, oxidative stress responses, inflammation, and fibrosis, thereby influencing MASLD onset and progression. Moreover, epigenetic dysregulation has also been implicated in hepatocarcinogenesis in the context of MASLD (Li et al., 2025). Epigenetics refers to molecular mechanisms that control gene expression without altering the DNA sequence and is classically divided into two major categories: DNA methylation, mediated by DNA methyltransferases (DNMTs) and ten–eleven translocation (TET) enzymes; and histone post-translational modifications, involving enzymes such as histone acetyltransferases (HATs), histone deacetylases (HDACs), and histone lysine demethylases (KDMs) (Wu et al., 2023).

Under physiological conditions, epigenetic alterations are essential for maintaining cell differentiation and identity (Rodríguez-Sanabria et al., 2022). However, under pathophysiological conditions such as metabolic dysfunction, alterations in epigenetic regulation can act as driving mechanisms that influence the initiation and progression of metabolic diseases (Chen and Natarajan, 2022; Wu et al., 2023). Experimental evidence from *in vitro* and *in vivo* models of diabetes, as well as observations in humans, demonstrates that epigenetic modifications induced by hyperglycemia are very persistent and feature a phenomenon known as metabolic memory, where metabolic dysfunction persists even after glucose levels normalized (Bansal and Simmons, 2018; Kato and Natarajan, 2019).

DNA methylation is one of the most extensively studied epigenetic mechanisms associated with metabolic dysfunction and related diseases (Wu et al., 2023). For example, diabetic conditions induced in a streptozotocin-treated zebrafish model resulted in global DNA hypomethylation, leading to aberrant gene expression patterns that were transmitted across cell divisions (Olsen et al., 2012; Chen et al., 2024). Similar findings have been confirmed in patients with type 2 Diabetes Mellitus, where hyperglycemia in poorly controlled patients seems to be negatively correlated with the global DNA methylation status (Pinzón-Cortés et al., 2017). In contrast, studies in cultured human hepatocytes have shown that lipid accumulation induced by high-glucose conditions increases DNA methylation at CpG motifs through upregulation of DNMT1 expression. Notably, hypermethylated genes were associated with 57 signaling pathways, including those directly involved in carbohydrate and lipid metabolism, such as PI3K, insulin, and cAMP-dependent pathways, as well as regulatory pathways such as Wnt signaling (Wang et al., 2020). These findings highlight a broad, yet context-dependent role of DNA methylation in metabolic dysfunction.

Analyses of human liver samples from MASLD patients at different disease stages further demonstrate that DNA methylation patterns correlate with disease progression, with distinct hypermethylated and hypomethylated regions. These epigenetic changes appear to be linked to chronic inflammation, oxidative stress, and accelerated epigenetic aging, all of which contribute to MASLD pathophysiology (Van Dijck et al., 2025). Similarly, global DNA hypomethylation has been observed in peripheral blood mononuclear cells (PBMCs) from patients with MASLD. In addition, elevated levels of 5-hydroxymethylcytosine (5hmC) have been detected in patients with moderate to high fibrosis risk (Barchetta et al., 2025), suggesting that DNA methylation-related marks may serve as important indicators of disease progression.

Emerging evidence also supports a role for mitochondrial DNA (mtDNA) hypermethylation as a key epigenetic signature in MASLD and a pivotal mechanism contributing to mitochondrial dysfunction (Theys et al., 2024). The experimental evidence linking mtDNA methylation to mitochondrial dysfunction in MASLD will be discussed in greater detail in subsequent sections.

Imbalances in histone post-translational modifications have also been described in metabolic diseases, including obesity, diabetes, and MASLD. Dysregulation of several histone modifications has been reported in MASLD, e.g., lysine methylation or demethylation at histone positions H3K4, H3K9, and H3K27, affecting genes involved in lipid synthesis and metabolism, oxidative stress responses, and inflammatory pathways (e.g., PPARγ, FASN, PNPLA3, SIRT1–NF-κB axis). Likewise, histone acetylation and deacetylation at e.g. H3K9, H3K27, and H4K8, regulate the expression of genes related to lipid synthesis and steatosis, including ChREBP, FASN, and SCD (Min et al., 2025). In general, histone acetylation is associated with transcriptional activation, whereas methylation at e.g., H3K9 and H3K27 is linked to transcriptional repression (Shi et al., 2023).

Studies in MASLD rat models have demonstrated hepatic downregulation of Enhancer of Zeste Homolog 2 (EZH2), a catalytic subunit responsible for H3K27 methylation. Similar findings have been observed in cultured hepatocytes under steatotic conditions (Vella et al., 2013), a mechanism that may exacerbate lipid accumulation and inflammation. In parallel, aberrant gene expression is a hallmark of metabolic dysfunction, with inflammatory transcription factors (e.g., NF-κB) and lipogenesis-related genes (e.g., ChREBP) being upregulated through increased HAT activity, particularly p300, which has been implicated in MASLD pathogenesis. Accordingly, both *in vitro* and *in vivo* studies have shown reduced lipogenesis following p300 inhibition (Bricambert et al., 2010; Lee et al., 2021). Furthermore, high-glucose conditions have been shown to induce persistent histone modifications that regulate genes involved in inflammation and antioxidant responses, reinforcing the concept of epigenetically mediated metabolic memory (Z. Chen and Natarajan, 2022).

Overall, this body of evidence underscores the relevance of epigenetic mechanisms as key drivers and modulators of MASLD pathophysiology (**Table 1**) and highlights their potential as therapeutic targets in the context of personalized medicine. **Table 1** summarizes most relevant experimental and translational studies focusing in the investigation of epigenetic mechanisms related to MASLD.

**Table 1:** Experimental and human studies investigating epigenetic modifications in MASLD.

Reference	Type of study	Model/sample	Epigenetic mechanism	Targets	Key findings	Relevance to MASLD
Bricambert et al., 2010	Experimental (*in vitro* & *in vivo*)	Hepatocyte cultures and animal models	Histone acetylation (HAT activity)	p300; lipogenic genes	Inhibition of p300 reduced hepatic lipogenesis	Identifies histone acetylation as a modifiable regulator of lipid metabolism
Olsen et al., 2012	Experimental (*in vivo*)	Streptozotocin-induced diabetic zebrafish	DNA methylation	Global DNA methylation	Diabetic conditions induced global DNA hypomethylation transmitted across cell divisions	Demonstrates persistent epigenetic changes underlying metabolic memory
Vella et al., 2013	Experimental (*in vivo* & *in vitro*)	MASLD rat model; steatotic hepatocytes	Histone methylation (H3K27)	EZH2	Downregulation of EZH2 reduced H3K27 methylation, exacerbating lipid accumulation and inflammation	Links histone methylation imbalance to MASLD pathogenesis
Wang et al., 2020	Experimental (*in vitro*)	Cultured human hepatocytes (high-glucose conditions)	DNA methylation (DNMT1 upregulation)	PI3K, insulin, cAMP, Wnt pathways	Increased CpG methylation and DNMT1 expression affect metabolic signaling	Highlights context-dependent DNA methylation in hepatic metabolic dysfunction
Lee et al., 2021	Experimental (*in vivo*)	Diet-induced MASLD animal models	Histone acetylation	p300; ChREBP	p300 inhibition reduced hepatic lipogenesis	Supports targeting histone acetylation in MASLD
Mposhi et al., 2023	Experimental/translational	Liver tissue and experimental models	mtDNA methylation	Mitochondrial genes	mtDNA hypermethylation is associated with mitochondrial dysfunction	Identifies mitochondrial epigenetics as a key MASLD mechanism
Cheng et al., 2025	Experimental (*in vitro* & *in vivo*)	High fat diet mice with 5-Aza-CdR treatment	DNA hypermethylation	AMPK signaling and PPAR-α, inflammatory genes	Reversing DNA hypermethylation and improves metabolic pathways but increases inflammatory gene expression	DNA differentially methylated regions marked potential therapeutic targets
Van Dijck et al., 2025	Human study	Liver biopsies from MASLD patients	DNA methylation	Inflammation, oxidative stress, and aging pathways	Disease-stage–specific methylation correlated with progression and epigenetic aging	Connects epigenetic remodeling with MASLD severity
Barchetta et al., 2025	Human study	PBMCs from MASLD patients	DNA methylation/hydroxymethylation	Global methylation; 5hmC	Global hypomethylation and increased 5hmC with fibrosis risk	Supports DNA methylation marks as non-invasive biomarkers
Min et al., 2025	Experimental/mechanistic	MASLD-relevant models	Histone acetylation & methylation	ChREBP, FASN, SCD	Histone modifications regulate lipid synthesis and steatosis	Defines chromatin-based regulation of hepatic lipid metabolism

In the following sections, we focus on the role of epigenetic regulation in cellular stress responses during MASLD development and progression, and we explore translational perspectives on the use of epigenetic modifiers for the design of novel therapeutic strategies.

## 3 Epigenetic response and cellular stress response in MASLD

### 3.1 Oxidative stress-induced epigenetic modifications

Epigenetic regulation has been increasingly recognized as a fundamental biological mechanism enabling cellular adaptation to diverse forms of stress. Oxidative stress, metabolic stress, and inflammatory stress (key drivers of MASLD pathogenesis) are closely linked to epigenetically mediated changes in gene expression (Chakraborty et al., 2025; Wu et al., 2024). These alterations can result in either global or locus-specific hypomethylation or hypermethylation of genes involved in stress responses. In this context, oxidative stress (OxS) has been associated with global DNA hypomethylation in several pathological conditions, including cancer, cardiovascular diseases, and aging. However, OxS can also induce localized hypermethylation of specific genomic regions, leading to gene silencing (Al-Awar and Hussain, 2024; Wu et al., 2024).

**Figure 1:**
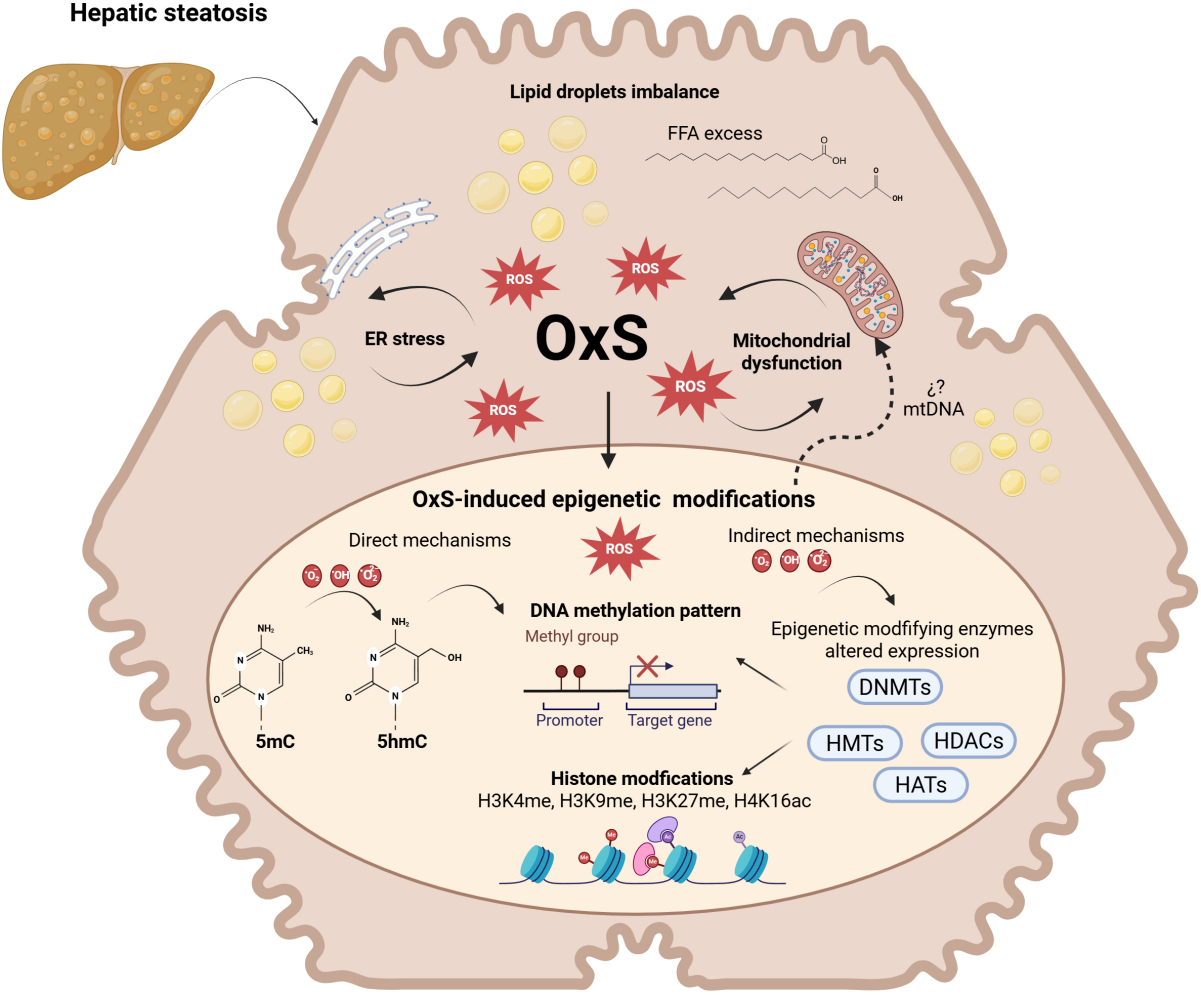
Epigenetic mechanisms related to cellular stress response during metabolic dysfunction-associated steatotic liver disease. Hepatic steatosis is characterized by lipid droplet accumulation and excess free fatty acids (FFAs), leading to endoplasmic reticulum (ER) stress and mitochondrial dysfunction. These alterations promote increased reactive oxygen species (ROS) production and oxidative stress (OxS), which further exacerbates cellular damage. OxS can induce epigenetic modifications through both direct and indirect mechanisms. Direct effects include ROS-mediated oxidation of DNA bases, influencing DNA methylation dynamics, such as the conversion of 5-methylcytosine (5mC) to 5-hydroxymethylcytosine (5hmC). Indirect mechanisms involve altered expression or activity of epigenetic-modifying enzymes, including DNA methyltransferases (DNMTs), histone methyltransferases (HMTs), histone acetyltransferases (HATs), and histone deacetylases (HDACs). Together, these processes lead to changes in DNA methylation patterns and histone modifications (e.g., H3K4me, H3K9me, H3K27me, H4K16ac), ultimately affecting gene expression and contributing to MASLD progression. Created in BioRender. Mancera (2026) https://BioRender.com/xlt0du8.

Oxidative stress is a central mechanism underlying organelle dysfunction and lipotoxicity in MASLD. In this context, production of reactive oxygen species (ROS) exceeds the antioxidant capacity of the liver, thereby driving cellular dysfunction, activation of proinflammatory signaling cascades, and progressive liver injury (Arroyave-Ospina et al., 2021). Accumulating evidence indicates that OxS induces epigenetic alterations through both direct and indirect mechanisms (**Figure 1****),** as demonstrated in studies investigating OxS-related aging and epigenetic regulation (Z. Wu et al., 2024; Chen et al., 2025; Ma et al., 2025). For nuclear DNA, global and locus-specific changes at CpG sites are associated with MASLD severity and inflammation under chronic exposure to free fatty acids and oxidized lipids (Romeo et al., 2025; Zhang et al., 2025). Also DNA methylation patterns in immune cells correlate with metabolic alterations in MASLD, positioning DNA methylation as an important mechanism, and potential biomarker (Romeo et al., 2025).

Directly, ROS can oxidize 5-methylcytosine (5mC) to 5-hydroxymethylcytosine (5hmC) by targeting cytosine residues present in CpG islands. ROS also induces chemical modifications of neighboring guanine bases (e.g., hydroxylation or alkylation) resulting in the formation of products such as -oxo-7,8-dihydroguanine (8oxoG) (Madugundu et al., 2014). These changes alter DNA methylation patterns by interfering with DNMT-dependent methylation processes and gene silencing (Madugundu et al., 2014). Indirectly, ROS modulates the expression and activity of epigenetic enzymes involved in DNA methylation (DNMT1, DNMT3A, DNMT3B, and MBD4) and histone modifications (HDAC1, HMT1, and HAT1). Among these, histone methylation and acetylation appear to be particularly sensitive to redox imbalance (Chen and Shen, 2025; Kumar et al., 2017; Vrtačnik et al., 2018). ROS levels also influence the activity of ten–eleven translocation (TET) enzymes in a context-dependent manner. High ROS levels are generally associated with reduced TET expression and limited cofactor availability, resulting in decreased DNA demethylation activity. Conversely, low or transient ROS levels may promote TET activation and expression (Delatte et al., 2015; Zuo et al., 2023). Similarly, ROS-driven upregulation of DNMT expression and activity—mediated in part by hypoxia-inducible factor α (HIF-α)—has been shown to induce hypermethylation and silencing of antioxidant response genes (Humphries et al., 2023). Consistent with this, increased expression of DNMT1, DNMT3A, and DNMT3B has been observed during OxS and aging, contributing to persistent gene silencing and representing potential therapeutic targets in OxS-related diseases (Bhargavan et al., 2024), which can be potentially targeted and may be useful as a therapeutic strategy in OxS-related diseases.

In contrast, ROS can also suppress DNMT expression and reduce the availability of the methyl donor S-adenosylmethionine (SAM), leading to global DNA hypomethylation (Al-Awar and Hussain, 2024; Huang et al., 2022). These apparently opposing effects highlight the complexity of OxS-mediated epigenetic regulation, which is highly dependent on factors such as ROS magnitude, exposure duration, cellular context, and disease stage (e.g., acute versus chronic stress, early versus advanced pathology).

ROS production itself is regulated by multiple ROS-generating enzymes, including NADPH oxidase 4 (NOX4), whose expression is epigenetically controlled. Studies in human fibroblast cultures have demonstrated enrichment of the H4K16 acetylation at the NOX4 promoter, correlating with increased gene expression and elevated ROS production during cellular senescence (Sanders et al., 2015). In parallel, epigenetic repression of antioxidant response genes (such as SOD2, SOD3, and NRF) has been reported in several *in vitro* aging models. These changes are mediated by DNMT3A-dependent DNA hypermethylation and increased H3K27 methylation, resulting in impaired antioxidant defenses (Jung et al., 2020; Roman et al., 2017; Thiruvengadam et al., 2021). Notably, this regulatory network appears to be bidirectional: histone methylation at H3K4 and H3K9 can further promote ROS overproduction by repressing NRF2 target genes while enhancing the expression of pro-oxidant genes, including NF-κB–dependent inflammatory mediators (Paneni et al., 2015).

Collectively, these findings underscore the intricate and context-dependent interplay between oxidative stress and epigenetic regulation in metabolic dysfunction. In MASLD, OxS-driven epigenetic remodeling might contribute to sustained alterations in gene expression that reinforce mitochondrial dysfunction, inflammation, and disease progression.

Therefore, organelle dysfunction during cellular stress response is an important driver mechanism in MASLD pathogenesis (Liu et al., 2025), which is likely regulated by epigenetic mechanisms, especially at the mitochondrial level, and targeting these mechanisms might be a reliable therapeutic approach in MASLD, as will be discussed in the next sections. Moreover, understanding the temporal and mechanistic determinants of these epigenetic responses will be essential for identifying actionable biomarkers and developing targeted therapeutic strategies aimed at modulating cellular stress responses in MASLD.

### 3.2 Epigenetic regulation and mitochondrial dysfunction

Next to epigenetic reprogramming, MASLD is driven by mitochondrial dysfunction (Zheng et al., 2023). Indeed, mitochondria are the main source of ROS production inside cells, a feature explaining the central role of mitochondrial dysfunction in MASLD. Likewise, mitochondrial DNA (mtDNA) is a main target for oxidative damage induced by ROS, and it has been demonstrated that ROS-derived products like 8-hydroxy-2-deoxyguanosine (8-OHdG) accumulate primarily in mtDNA during OxS conditions, which might result in methylation changes (Yue et al., 2022; Tian et al., 2024). Due to its biological relevance, epigenetic regulation in the context of mitochondrial dysfunction and its implications in MASLD will be discussed in the next section.

**Figure 2:**
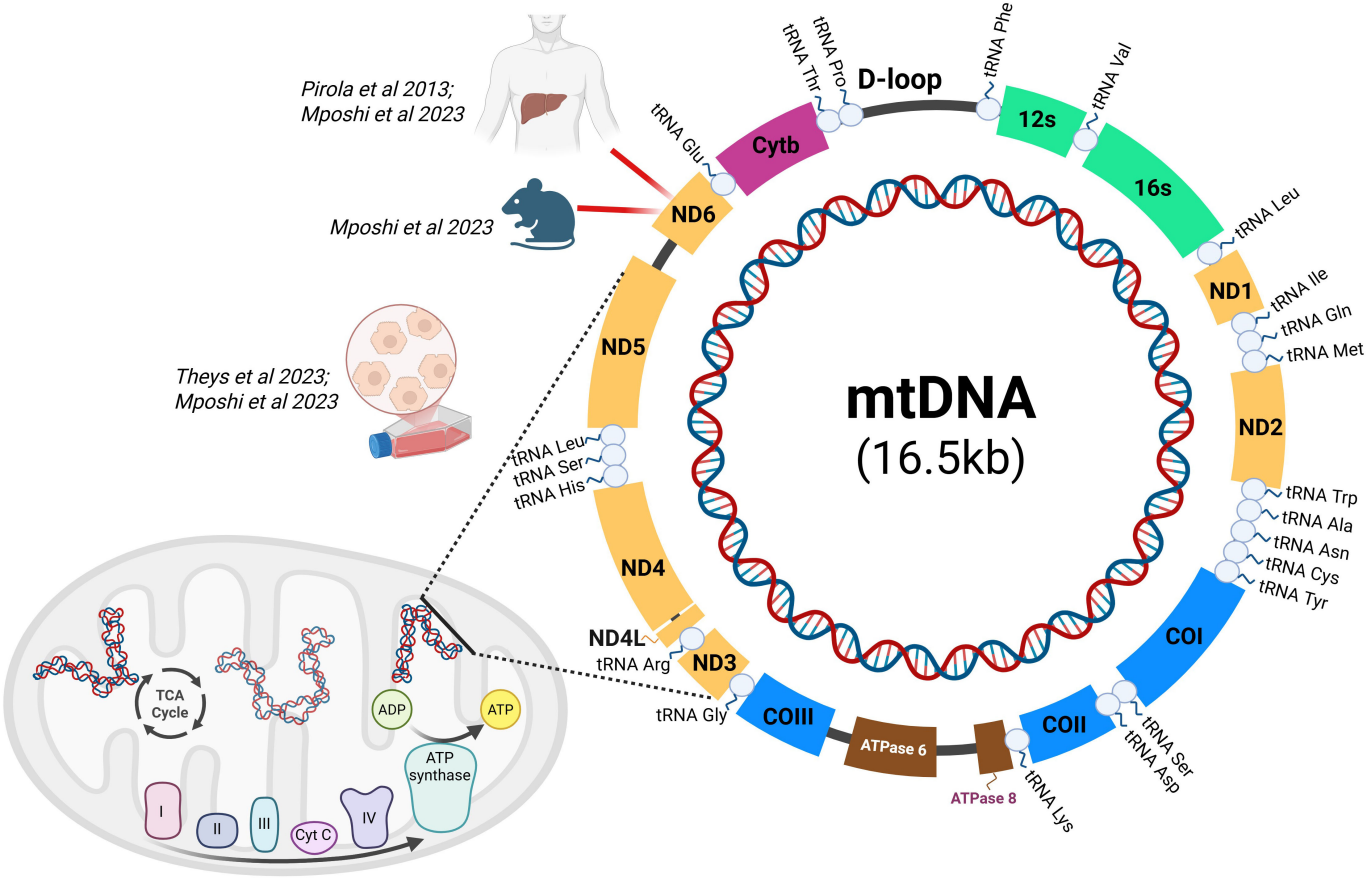
mtDNA methylation findings in hepatic steatosis models. Mitochondrial DNA-encoded genes findings in transgenic HepG2 cells, mice and patients. Schematic representation of the human mitochondrial genome (mtDNA, 16.5 kb) and mtDNA-encoded genes reported to be epigenetically altered in hepatic steatosis. The transgenic HepG2 MSssI and MCviPI cell lines, which overexpress a CpG-specific bacterial and viral GpC DNMT, respectively, both showed an increased overall CpG/GpC methylation (~20%), compared to control cells (~0.04-0.4%) (Mposhi et al., 2023; Theys et al., 2023), while studies with mice and human liver samples revealed higher mtDNA CpG methylation at ND6 gene (Pirola et al., 2013, Mposhi et al., 2023. Studies showed changes in mtDNA methylation affecting genes encoding subunits of the oxidative phosphorylation system, including ND genes, cytochrome b (Cytb), cytochrome c oxidase subunits (COI–COIII), and ATP synthase subunits. Alterations in mtDNA methylation are associated with impaired mitochondrial function, altered electron transport chain activity, and reduced ATP production, contributing to metabolic dysfunction in hepatic steatosis. Created in BioRender. Mancera (2026) https://BioRender.com/xlt0du8.

Mitochondria are fundamental players in metabolism, energy homeostasis, and molecular signaling. These organelles are involved in glucose and lipid metabolism, and ATP production by means of the tricarboxylic acid (TCA) cycle and oxidative phosphorylation (Caputo et al., 2023). The dysfunction of these organelles by increased ROS production disrupts mitochondrial membrane potential, ATP generation, and reduces their biogenesis (Caputo et al., 2023; Zheng et al., 2023). Mitochondrial dysfunction is strongly associated with MASLD because of its role in lipid metabolism. Factors such as mitophagy, oxidative stress, differentiation, and biogenesis quality control differentially influence mitochondrial function to promote liver fat accumulation and injury (Zheng et al., 2023). DNA methylation, histone post-translational alterations and chromatin remodeling can modulate transcriptional networks controlling lipid metabolism, inflammation, and mitochondrial genes in steatosis. Reviews and targeted studies document altered mitochondrial nucleotide methylation and sirtuin-regulated histone deacetylation in MASLD. In addition, methylation of mitochondrial DNA (mtDNA) has been described *in vitro*, in mouse models, and in patients (Mposhi et al., 2023; Pirola et al., 2013; Theys et al., 2023), as observed in **Figure 2**. In fact, mtDNA hypermethylation impaired gene expression and mitochondrial metabolic activity in HepG2 cells, leading to increased lipid accumulation (Mposhi et al., 2023; Theys et al., 2023). In addition, alterations in ND6 DNA methylation and transcription were observed in mice under high-fat, high-cholesterol diets, and in human liver samples, suggesting mtDNA methylation may alter mitochondrial transcription and function (Mposhi et al., 2023). Additionally, transcriptional analysis of transgenic HepG2 cells expressing mtDNA-targeted DNA methyltransferase showed that hypermethylation correlates with upregulation of bile acid metabolism genes, including NR5A2 and PPARα nuclear receptors (Theys et al., 2023). Evidence of mitochondrial swelling increased mitochondrial respiration, and the activation of the mitophagy/autophagy stress response was observed. Additional findings in a sea fish model (Larimichthys crocea) fed with dietary lipids like olive oil and perilla oil were associated with hypermethylation of mtDNA regions encoding NADH dehydrogenase subunits (MT-ND4L) and transfer RNA (MT-TR), which correlated with altered expression of these mitochondrial genes (Caputo et al., 2023). High dietary lipid content also led to hypermethylation of the D-loop region and hypomethylation of the 12S ribosomal RNA gene (MT-RNR1) from liver mtDNA. These studies suggest a potential role for mtDNA methylation in mitochondrial dysfunction and lipid metabolism in MASLD, but the exact mechanisms remain to be elucidated.

Additional ways in which epigenetic alterations modulate mitochondrial function in conditions of lipid stress are linked to histone modifications at nuclear-encoded genes. This is the case of NAD+/dependent sirtuin deacetylases (SIRT) that regulate histone and non-histone acetylation states to influence PGC 1α, SREBP 1c, and other metabolic transcription factors relevant to mitochondrial biogenesis and lipid metabolism (La Colla et al., 2023). Sirtuins link cellular NAD+ status to chromatin state and mitochondrial biogenesis, providing a mechanistic bridge between cellular energy/redox state and epigenetic control of metabolic genes (Jiang et al., 2021; Shen et al., 2024). Epigenetic deacetylases (SIRT1) deacetylate PGC-1α and other transcription factors to promote mitochondrial biogenesis and β-oxidation, and disruption of this mechanism (for example via IGF2 knockdown, reducing SIRT1/PGC-1α during liver fat accumulation), impairs mitochondrial biogenesis and increases ROS in hepatocytes (Jiang et al., 2021).

## 4 Lifestyle and epigenetic interventions in MASLD

Lifestyle interventions in MASLD, including dietary patterns, physical activity, and other environmental factors, are critically influencing disease progression. Therefore, diet interventions by reducing saturated fats (e.g., simple sugars and processed food) and increasing physical activity have shown a beneficial correlation with weight loss and visceral fat loss, resulting in MASLD improvement (Chen et al., 2025). Recent evidence demonstrated that a healthy lifestyle in MASLD patients results in a significant reduction of steatosis grade, which correlates with improvement of lipid metabolism and inflammatory response (Wang et al., 2025). Likewise, diet and exercise can improve liver fibrosis and transaminase in MASLD patients, often associated with a sustained weight loss (Desai & Hajare, 2025), demonstrating that lifestyle-based interventions are effective therapeutic approaches in MASLD.

As mentioned, unhealthy diet and lack of physical activity are considered the main lifestyle factors associated with MASLD; however, a change to a healthy lifestyle is an effective measure to control, and even reverse, early stages of liver steatosis (Wang et al., 2025). It is known that lifestyle can strongly affect epigenetic mechanisms in the development of liver steatosis (da Costa Fernandes et al., 2026; Liang et al., 2024). As an example, dietary interventions can modify DNA methylation patterns, linked to locus−specific hypermethylation in high-fat diet models (Tong et al., 2019) or genome−wide changes as observed in human diet trials (Sokolowska et al., 2022). In this context, Liang and coworkers investigated the effects of supplementation of vitamin D (VD) on the occurrence of MASLD and liver fibrosis (Liang et al., 2024). In a high-fat diet-induced (HFD) mouse model, VD was supplemented (10.000 IU/kg) for 20 weeks. Compared to the HFD mice control, the VD-supplemented group showed lower expressions of genes related to inflammation, lipogenesis, and lipid oxidation in the liver, and significantly reduced total cholesterol, AST, and ALT levels. Histological analysis confirmed that vitamin D supplementation ameliorated hepatic inflammation, ballooning, steatosis, and total pathological scores. Inhibition of DNMT1 expression reverses the epigenetic patterns on the VD metabolism genes and TGFbR1 also *in vitro*, which ultimately triggered the TGFb1/Smad3 pathway to result in the development of fibrosis in MASLD. The authors reported that VD-dependent protective effects were weakened by the treatment with gene silencing of DNMT1 as well. To investigate the impact of physical exercise on liver tissue in obese animals, male Swiss mice under high-fat diet conditions were divided into Control (CT), Sedentary Obese (SOB), and Trained Obese (TOB) groups (da Costa Fernandes et al., 2026; C. Wu et al., 2025). Strength exercise reduced fasting glycemia, restored hepatic insulin signaling, enhanced mitochondrial biogenesis and oxidative capacity, and increased ATP5 protein expression. Exercise trained mice showed in liver an epigenetic modulation of the MTCH2 (Mitochondrial carrier homolog 2) promoter region that encodes a protein localized on the outer mitochondrial membrane to assist in the recruitment of the proapoptotic BID (The BH3-interacting domain death agonist) protein (Wu et al., 2025). Strength exercise decreased DNA methylation in the MTCH2 promoter region, which correlated with increased MTCH2 mRNA expression in TOB groups. Finally, in the trained obese mice, a reduction in DNMT1 mRNA was observed, but an increase in TET1 and TET3. In summary, this work shows that training interventions can reverse obesity- related epigenetic mechanisms.

An *in vivo* study using a mouse model explored the impact of decreasing global DNA methylation via 5-Aza-CdR epidrug on MASLD (Cheng et al., 2025). In this work, mice were fed with a high-fat diet (HFD) for 9 weeks: before pregnancy, during pregnancy, and lactation. After weaning, male offspring were split into HFD groups w/o 5-Aza. The 5-AZA+ HFD group exhibited reduced weight gain, improved glucose regulation, and minimized hepatic fat buildup and serum lipid imbalances compared to the HFD. Also, male mice significantly reduced global DNA methylation levels and DNMT activity in the liver, without affecting the expression of Dnmt1, Dnmt3a, or Dnmt3b. Differentially methylated regions (DMRs) in the promoters of Bax, Casp3, and Il6, which were associated with changes in the expression of these genes as well (Cheng et al., 2025). The 5-AZA-treated mice demonstrated increased activation of AMPK signaling and increased PPAR-α expression, and levels of methylation correlated with upregulation of proapoptotic genes (Bax, Casp3, Ifn-γ), proliferative genes (Atm, Cdk2, Cdk4, CycD2), and pro-inflammatory cytokines (Il-1β, Il-6, TNF-α) compared to the HFD group. According to this data, reversing liver DNA methylation using epidrugs can prevent MASLD in offspring exposed to maternal overnutrition, suggesting a potential preventive strategy for managing these chronic metabolic conditions. However, the increased hepatic inflammation associated with 5-AZA treatment underscores the complexity of epigenetic modulation and its dual impact on metabolic health. Other epigenetic modifications, particularly histone acetylation regulated by histone deacetylases (HDACs), have emerged in the development and progression of liver steatosis (Shi et al., 2023). Trichostatin A (TSA), a potent reversible inhibitor of class I and II HDACs, has been used as epidrug to investigate the role of this epigenetic mark in liver steatosis. Chromatin immunoprecipitation (ChIP) analysis revealed that TSA-induced histone hyperacetylation occurs at promoter regions of metabolically important genes, including carnitine palmitoyltransferase 1α (Cpt1α), a rate-limiting enzyme in fatty acid β-oxidation (Kirpich et al., 2013). By preventing HDAC3 action and enhancing H3K9 acetylation at the Cpt1α promoter, TSA upregulates transcription of this critical metabolic gene, increasing fatty acid oxidation and reducing hepatic lipid accumulation (Ellis et al., 2009; Kirpich et al., 2013). TSA also influences mitochondrial function and energy metabolism in hepatocytes. High-fat diet studies in MASLD models have demonstrated that HDAC inhibition affects mitochondrial turnover through restricted acetylation, suggesting complex interactions between nuclear epigenetic regulation and mitochondrial dynamics (Aghayev et al., 2023). Hepatic stellate cells (HSCs) are the primary effectors in liver fibrogenesis, and TSA has been shown to inhibit their activation (Chen et al., 2015; Ding et al., 2018). Specifically, TSA increases C/EBP-α gene acetylation both *in vivo* and *in vitro* (Ding et al., 2018). As C/EBP-α is a transcription factor that prevents hepatic stellate cell (HSC) division (G0 state), its hyperacetylation keeps these cells in a quiescent state and prevents their transformation into myofibroblast-like cells that produce excessive extracellular matrix (Ding et al., 2018). Also, TSA affects the expression of α-smooth muscle actin (αSMA) and collagen-1, key markers of HSCs activation and fibrogenesis (Samuvel et al., 2024). Additionally, there is evidence that specific medication can enhance certain epigenetic marks in hepatic steatosis. To study the effect of the drug pirfenidone (PFD: 5-methyl-1-phenyl-2(1-H)-pyridone) as an epigenetic regulator (H3K9 methylation), male C57BL/6J mice under high fat/carbohydrate diet were treated (Rodriguez-Sanabria et al., 2024). PFD is an antifibrogenic molecule approved for the treatment of idiopathic pulmonary fibrosis by the Food and Drug Administration. Here, PFD-treated mice showed reduced weight gain, epididymal fat, inflammatory nodules, macrosteatosis, and fibrosis in liver tissue, as well as improved biochemical tests: AST/ALT, glucose, cholesterol, triacylglycerides and VLDL. At the molecular level, the PDF-treated group showed reduced JMJD2B protein expression and increased H3K9me3 repressive marks in the promoter regions of FASN, SREBP1, and PPARg genes, as well as across the whole genome. Additional *in vitro* experiments in HepG2 cells also demonstrated reduced lipid accumulation, correlating with the reduction of JMJD2B and an increase in the H3K9me3 epigenetic mark. Rodriguez-Sanabria and coworkers thus demonstrated how e new epidrugs can modulate H3K9me3 deposition and ultimately reduce MASH marks.

**Figure 3:**
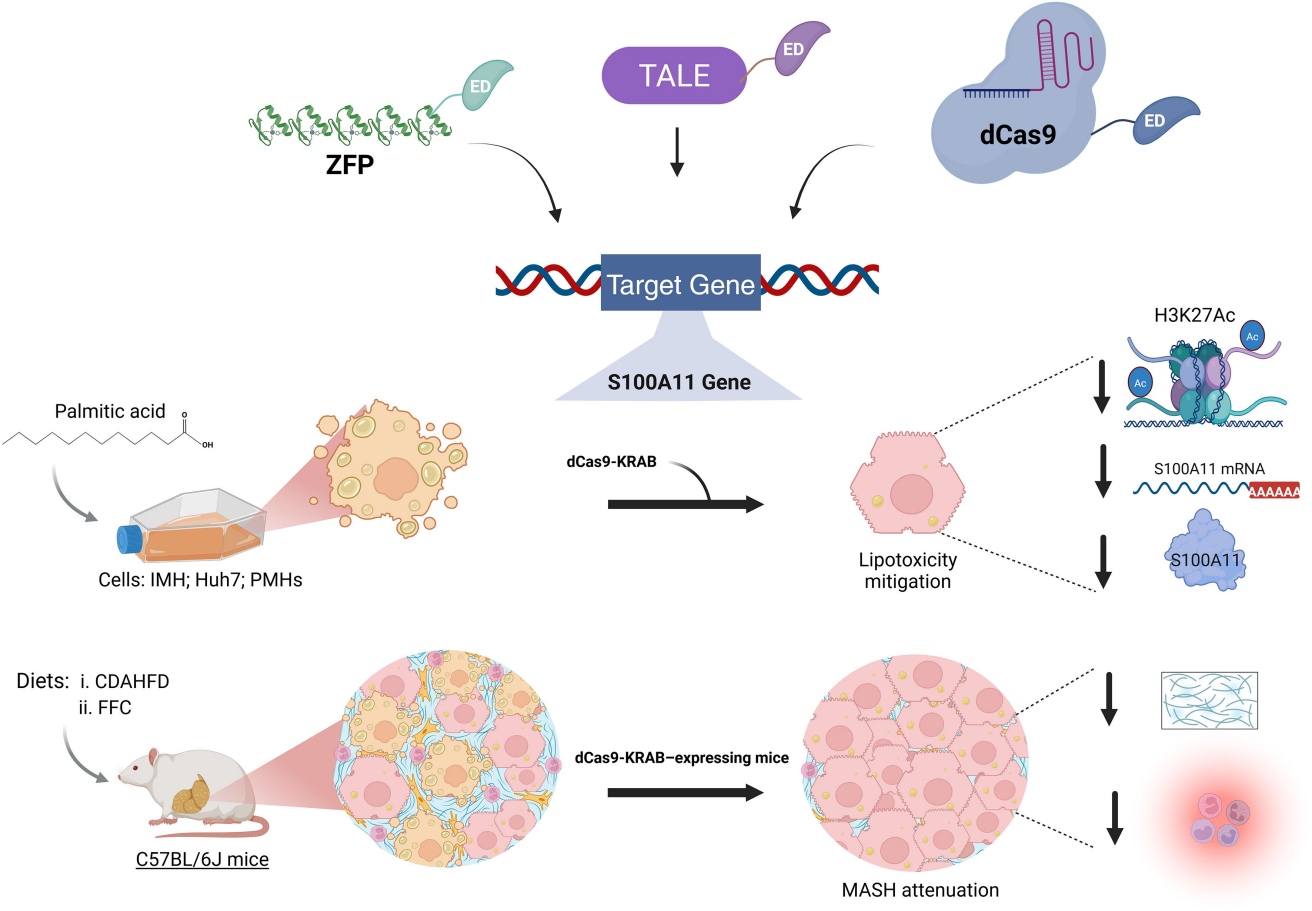
Epigenetic editing platforms. Schematic representation of programmable epigenetic editing technologies based on zinc finger proteins (ZFP), transcription activator-like effectors (TALEs), and catalytically inactive CRISPR-associated protein 9 (dCas9) at the top. These platforms enable sequence-specific targeting of genomic loci through engineered DNA-binding domains (ZFP, TALE) or guide RNA–directed recognition (dCas9). When coupled to epigenetic effector domains (ED), they allow precise modulation of gene expression by inducing targeted epigenetic modifications without altering the underlying DNA sequence (Cortés-Mancera et al., 2022). A pioneering study in MASLD used dCas9 technology for targeting a transcriptional repressor (KRAB) to a lipotoxicity-influenced enhancer [shown at the bottom (Daniel et al., 2025)]. Lipotoxicity was induced by palmitic acid in immortalized, non-transformed, wild-type hepatocyte cell line (IMH), the liver cell line Huh7, and primary mouse hepatocytes (PMHs) before targeting the S100A11 enhancer region. For the *in vivo* model (C57BL/6J mice), two diet regimens were implemented: Choline-deficient, L-amino acid-defined high-fat diet (CDAHFD) and High-fat, high-fructose, and high-cholesterol (FFC). A decrease in histone 3, lysine 27 acetylation marks (H3K27Ac) was observed *in vitro*, alongside a reduction of S100A11 mRNA and protein expression upon dCas9-KRAB treatment. Experiments in a genetically modified animal model (dCas9-KRAB expressing mice) showed a reduction in fibrosis, collagen deposition, and liver inflammation. Created in BioRender. Mancera (2026) https://BioRender.com/xlt0du8.

More recently, epigenetic editing technologies have emerged as powerful tools to interrogate and potentially reprogram disease-associated epigenetic states and are currently being tested in clinical trials (Heller et al., 2026). As shown in **Figure 3**, these approaches take advantage of programmable DNA-binding platforms CRISPR/dCas9 (catalytically deactivated Cas9), zinc finger proteins (ZFPs), and transcription activator-like effectors (TALEs)—fused to epigenetic effector domains (Epi-editors) that can write or erase chromatin marks at specific genomic loci (Cortés-Mancera et al., 2022).

Unlike traditional genome editing that permanently alters DNA sequences, epigenetic editing modulates gene expression through reversible chromatin modifications without introducing genotoxicity, offering potential advantages for treating complex metabolic diseases where fine-tuned gene regulation is required (Cortés-Mancera et al., 2022; Heller et al., 2025). Among the battery of potential gene expression modulating effector domains, the KRAB, DNMT3A/3L and TET 1 DNA demethylase catalytic domain (TET1CD) fused to dCas9 protein have been used to target genes involved in hepatic steatosis (Thakore et al., 2018; Hanzawa et al., 2020; Cappelluti et al., 2024; Daniel et al., 2025; Tremblay et al., 2025). The KRAB domain passively recruits KAP1 and histone-modifying enzymes to establish repressive heterochromatin (Thakore et al., 2018), while TET1 oxidizes 5-methylcytosine to 5-hydroxymethylcytosine, initiating active DNA demethylation (Cortés-Mancera et al., 2022). In the context of liver diseases, the dCas9 system has also been applied to repress the proinflammatory Alarmin S100A11 (S100 calcium-binding protein A11) enhancer, which is upregulated by lipotoxic ER stress (Palmitate stimulation), leading to increased H3 lysine 27 acetylation (H3K27ac) (Daniel et al., 2025). This work represents a proof-of-concept for targeting disease-specific lipotoxicity-influenced enhancers in MASLD/MASH context (Daniel et al., 2025). The authors demonstrated histone-mediated repression specifically reduced palmitate-induced H3K27ac in cultured hepatocytes upon dCas9-KRAB administration, and provided validation in two different mouse models, achieving significant reduction in S100A11 expression with downstream inflammatory responses, reduced liver injury, less fibrosis, and collagen deposition. On the other hand, Hanzawa et al. employed dCas9-SunTag-TET1CD to demethylate the Fgf21 (Fibroblast growth factor 21) promoter in Hepa1–6 liver cells and PPARα-deficient mice, demonstrating that targeted demethylation of Fgf21 enhances gene expression response to PPARα activation and fasting (Hanzawa et al., 2020). Although the transient approach did not show significant differences in methylation or Fgf21 gene expression longer than 14 days, this study provides direct evidence that DNA methylation status determines the magnitude of gene expression responses to metabolic contexts. Similarly, Cappelluti and coworkers targeted the Pcsk9 (Proprotein convertase subtilisin/kexin type 9) gene, which controls circulating levels of cholesterol by promoting the degradation of the low-density lipoprotein (LDL) receptor on the plasma membrane of hepatocytes (Cappelluti et al., 2024; Tremblay et al., 2025). Here, “hit-and-run” epigenetic editing was performed to induce long-lasting gene modulation using transient mRNA delivery of engineered transcriptional repressors (ETRs): KRAB, cdDNMT3A and DNMT3L. These studies convincingly show that epigenetic editing can be equally effective as Cas9 wildtype-mediated gene editing and that silencing can be maintained for over a year, even upon partial hepatectomy. Moreover, the silencing effect was fully reversible by targeting TET1, demonstrating the power of epigenetic editing.

Delivery systems for CRISPR editing technology are still a challenge for translational epigenetics (Heller et al., 2025). Although Adeno-associated viruses (AAVs) and lentiviral vectors (LVs) are commonly applied for delivering CRISPR components (Jung et al., 2025; Lučanský et al., 2023), both have limitations. AAVs packaging capacity limits the size of cargo, in addition, pre-existing immunity to AAV serotypes can decrease delivery efficiency (Lučanský et al., 2023; Pradhan and Anoop, 2026). On the other hand, LVs present larger packaging capacity but integrate into the host genome, interrogating safety conditions for wide applications (Lučanský et al., 2023). Among non-viral delivery systems, recently Lipid nanoparticles (LNPs) have emerged as a promising strategy, specifically for mRNA-based therapeutics (Feehley et al., 2023; Cappelluti et al., 2024; Heller et al., 2025). This novel delivery technology can carry mRNA encoding dCas-fusions along with guide RNA sequences, enabling transient expression and reducing the risk of prolonged off-target effects (Feehley et al., 2023). In fact, LNPs have been used to effectively deliver CRISPR-dCas9 to the liver in preclinical studies (Cappelluti et al., 2024; Tremblay et al., 2025) and are currently exploited in clinical trials to treat hepatitis B-infected patients (Fonseca et al., 2026). Tremblay and coworkers evaluated the capacity of a dual epi-editor fused to dCas9 (DNMT3A/3L-dCas9-KRAB and dCas9-TET) for long-lasting silencing (and its reversal) of PCSK9 gene, which plays a role in the liver’s cholesterol regulation, as previously mentioned. In primary human hepatocytes, the LNPs delivery led to a large increase in methylation with specificity demonstrated by Whole-Genome bisulfite sequencing, while targeting a PCSK9-Tg mice model reduced PCSK9 blood stream circulation in 98% with at least 1 year of durability (Tremblay et al., 2025). The administration of the DNMT3A/3L-dCas9-KRAB epi-editor in non-human primates (Cynomolgus monkeys) by these authors led to a rapid and durable reduction in circulating PCSK9 (90%) and LDL-cholesterol (70%). These findings demonstrate the therapeutic potential, efficiency and durability of epigenetic editing *in vivo*, which upon targeting the opposite effector (TET) was reversible. However, achieving efficient delivery to specific tissues beyond the liver remains challenging (Heller et al., 2025).

## 5 Discussion and perspectives

MASLD has emerged as a major global health challenge, driven by its close association with cardiometabolic dysfunction and its rapidly increasing prevalence worldwide. Although substantial progress has been made in identifying genetic variants associated with disease susceptibility, it has become increasingly evident that genetic predisposition alone cannot account for the marked heterogeneity observed in MASLD onset, severity, and progression. In this context, epigenetic regulation has gained prominence as a central mechanistic layer integrating genetic background with environmental, metabolic, and lifestyle-related exposures.

Epigenetic mechanisms are dynamic regulators of hepatic metabolic homeostasis and cellular stress responses in MASLD. DNA methylation, histone post-translational modifications, and chromatin remodeling collectively orchestrate transcriptional programs governing lipid metabolism, oxidative stress responses, inflammation, mitochondrial function, and fibrogenesis. Importantly, these epigenetic alterations are not merely passive consequences of metabolic dysfunction but actively contribute to disease initiation and progression, reinforcing maladaptive stress responses, and promoting metabolic memory.

A key concept emerging from the current literature is the tight interplay between epigenetic remodeling and cellular stress pathways. Oxidative stress, ER stress, and mitochondrial dysfunction are central drivers of hepatocellular injury in MASLD and are, in turn, modulated by epigenetic mechanisms. ROS-induced changes in DNA methylation and histone modifications can silence antioxidant defenses, amplify inflammatory signaling, and perpetuate mitochondrial dysfunction, thereby establishing self-reinforcing pathogenic loops. The context-dependent nature of these epigenetic responses—shaped by stress intensity, duration, and disease stage—underscores the complexity of MASLD pathophysiology and highlights the need for temporally resolved and cell-type–specific analyses.

Mitochondrial dysfunction represents a particularly compelling intersection between metabolic stress and epigenetic regulation. Emerging evidence supports a role for both nuclear and mitochondrial epigenetic modifications in shaping mitochondrial gene expression, bioenergetic capacity, and lipid handling. Alterations in mtDNA methylation, alongside epigenetic regulation of nuclear-encoded mitochondrial regulators such as PGC-1α and sirtuins, link redox state and chromatin dynamics to mitochondrial biogenesis and quality control. While these findings position mitochondrial epigenetics as a promising mechanistic and therapeutic avenue, important questions remain regarding the regulation, functional relevance, and reversibility of mtDNA methylation in human MASLD.

Beyond mechanistic insights, epigenetic signatures hold significant translational potential as biomarkers for disease diagnosis, prognosis, and patient stratification. Differential DNA methylation patterns in liver tissue and peripheral blood cells correlate with disease severity, fibrosis risk, and accelerated epigenetic aging, suggesting that epigenetic profiling could complement existing clinical and imaging-based tools. The accessibility of peripheral epigenetic markers further supports their potential utility in non-invasive monitoring and longitudinal risk assessment.

From a therapeutic perspective, the reversibility of epigenetic modifications provides a strong rationale for targeting these pathways in MASLD. Lifestyle interventions, including diet and physical exercise, have demonstrated the capacity to remodel epigenetic marks associated with insulin signaling, mitochondrial function, and lipid metabolism. These findings reinforce the concept that behavioral interventions can exert durable molecular effects through epigenetic reprogramming. At the same time, pharmacological modulation of epigenetic enzymes using epidrugs has shown promising preclinical efficacy in reducing steatosis, inflammation, and fibrosis. However, the pleiotropic and sometimes opposing effects of global epigenetic modulation highlight the need for caution, as well as for the development of more selective epigenetic therapies, like epigenetic editing.

Looking ahead, several challenges must be addressed to fully harness the potential of epigenetic-based precision medicine in MASLD. These include improving mechanistic resolution through single-cell and spatial epigenomics, clarifying the temporal dynamics of epigenetic remodeling across disease stages, and disentangling causality from consequences in epigenetic changes. In addition, integrating epigenetic data with genomic, transcriptomic, metabolomic, and clinical information will be essential for defining robust molecular endotypes and guiding individualized therapeutic strategies.

In conclusion, epigenetic regulation represents a wide landscape linking metabolic stress, cellular dysfunction, and disease heterogeneity in MASLD. By bridging genetic susceptibility and environmental exposure, epigenetic mechanisms provide critical insights into disease pathogenesis while offering promising opportunities for biomarker development and targeted intervention. Continued translational and mechanistic research in this field will be pivotal for advancing precision-based approaches to MASLD prevention, diagnosis, and treatment.

## Glossary

5-AZA/5-Aza-CdR: 5-aza-2′-deoxycytidine (Decitabine)
5hmC: 5-hydroxymethylcytosine
5mC: 5-methylcytosine
8oxoG: 8-oxo-7,8-dihydroguanine
AMPK: AMP-activated protein kinase
CAV1: Caveolin 1
ChREBP: Carbohydrate response element-binding protein
CpG: Cytosine-Guanine dinucleotide
CPT1α: Carnitine palmitoyltransferase 1α
DNMTs: DNA Methyltransferases (DNA MTases)
ER stress: Endoplasmic reticulum stress
EZH2: Enhancer of Zeste Homolog 2
FASN: Fatty acid synthase
FFA: Free fatty acid
HATs: Histone acetyltransferases
HDACs: Histone deacetylases
HMTs: Histone methyltransferases
KDMs: Histone lysine demethylases
MASLD: Metabolic dysfunction-associated steatotic liver disease
mtDNA: Mitochondrial DNA
MT-ND4L: Mitochondrially Encoded NADH Ubiquinone Oxidoreductase Core Subunit 4L
MT-RNR1: 12S ribosomal RNA gene
MT-TR: Mitochondrially Encoded Transfer RNA
ND6: Mitochondrially Encoded NADH Ubiquinone Oxidoreductase Core Subunit 6
NOX4: NADPH oxidase 4
NR5A2: Nuclear receptor subfamily 5 group A member 2
OxS: Oxidative stress
PBMCs: Peripheral blood mononuclear cells
PGC 1α: Peroxisome proliferator-activated receptor gamma coactivator 1-alpha
PPARα: Peroxisome proliferator-activated receptor alpha (also known as NR1C1)
PPARγ: Peroxisome proliferator-activated receptor gamma
ROS: Reactive Oxygen Species
SAM: S-adenosylmethionine
SIRT/SIRT1: NAD-dependent deacetylase sirtuin-1
SREBP 1c: Sterol regulatory element-binding proteins isoform 1
TET: Ten–eleven translocation enzymes
TSA: Trichostatin A
VLDL: Very-low-density lipoprotein.
